# β-TrCP-Mediated Proteolysis of Mis18β Prevents Mislocalization of CENP-A and Chromosomal Instability

**DOI:** 10.1080/10985549.2024.2382445

**Published:** 2024-08-13

**Authors:** Subhash Chandra Sethi, Roshan Lal Shrestha, Vinutha Balachandra, Geetha Durairaj, Wei-Chun Au, Michael Nirula, Tatiana S. Karpova, Peter Kaiser, Munira A. Basrai

**Affiliations:** a Genetics Branch, National Cancer Institute, National Institutes of Health, Bethesda, Maryland, USA; b Department of Biological Chemistry, School of Medicine, University of California, Irvine, California, USA; c Laboratory of Receptor Biology and Gene Expression, National Cancer Institute, National Institutes of Health, Bethesda, Maryland, USA

**Keywords:** β-TrCP, E3 ubiquitin ligase, Mis18β, centromere, CENP-A, chromosomal instability

## Abstract

Restricting the localization of evolutionarily conserved histone H3 variant CENP-A to the centromere is essential to prevent chromosomal instability (CIN), an important hallmark of cancers. Overexpressed CENP-A mislocalizes to non-centromeric regions and contributes to CIN in yeast, flies, and human cells. Centromeric localization of CENP-A is facilitated by the interaction of Mis18β with CENP-A specific chaperone HJURP. Cellular levels of Mis18β are regulated by β-transducin repeat containing protein (β-TrCP), an F-box protein of SCF (Skp1, Cullin, F-box) E3-ubiquitin ligase complex. Here, we show that defects in β-TrCP-mediated proteolysis of Mis18β contributes to the mislocalization of endogenous CENP-A and CIN in a triple-negative breast cancer (TNBC) cell line, MDA-MB-231. CENP-A mislocalization in β-TrCP depleted cells is dependent on high levels of Mis18β as depletion of Mis18β suppresses mislocalization of CENP-A in these cells. Consistent with these results, endogenous CENP-A is mislocalized in cells overexpressing Mis18β alone. In summary, our results show that β-TrCP-mediated degradation of Mis18β prevents mislocalization of CENP-A and CIN. We propose that deregulated expression of Mis18β may be one of the key mechanisms that contributes to chromosome segregation defects in cancers.

## Introduction

Faithful chromosome segregation is a vital process that ensures equal distribution of genetic material to daughter cells during mitosis. The centromere is a specific locus on a chromosome where a specialized multiprotein structure called kinetochore assembles during cell division.^1^ The fundamental role of the kinetochore is to mediate the attachment of sister chromatids with the mitotic spindle apparatus during metaphase and eventually integrate spindle forces for proper chromosome separation during anaphase.^2^^,^^3^ Kinetochore establishment on the centromeric region is dictated by the presence of a histone H3 variant, CENP-A (centromere protein A) in humans, Cse4 in budding yeast, Cnp1 in fission yeast and Cid in flies. In most eukaryotes, both centromere formation and CENP-A deposition are controlled epigenetically by the presence of specific proteins at centromeric regions independent of the underlying DNA sequence.^4^ The CENP-A chromatin first recruits the constitutive centromere-associated network (CCAN) proteins, which have the ability to bind to the centromeric DNA, and then engages outer kinetochore proteins on the CCAN platform to orchestrate functional kinetochore assembly.^2^ Mutation or depletion of either CENP-A or CCAN proteins results in improper kinetochore assembly and chromosome segregation defects.^5–8^

During centromeric DNA replication in S-phase, CENP-A containing nucleosomes are quantitatively maintained at the centromeres owing to their high stability.^9–11^ The partitioning of CENP-A between the parental and newly synthesized daughter DNA strands is crucial to preserve the epigenetic identity of the centromeres and as a result, the centromeric levels of CENP-A undergo dilution, a process known as replicative dilution.^12^ However, histone H3.3 is known to be deposited concomitantly during DNA replication at the CENP-A chromatin domains and persist until new CENP-A loading is achieved.^12^ The replenishment of CENP-A in newly divided cells occurs precisely during late telophase or early G1 phase of the cell cycle.^13^ Hence, deposition and maintenance of CENP-A at the centromeres require spatiotemporal regulation by important regulatory proteins. Studies including genetic and biochemical analyses have uncovered such regulators in yeasts, worms, flies, and vertebrates. In fission yeast, mutants that show defects in chromosome segregation led to the identification of *mis16* and *mis18* mutations that caused the depletion of Cnp1 (fission yeast CENP-A) at the centromeres.^14^ Mammalian homologs of *Schizosaccharomyces pombe* Mis16, RbAp46 and RbAp48 function as histone chaperones and cells depleted for these proteins exhibit CENP-A assembly defects.^14^^,^^15^ Mis18 complex regulates the recruitment of Holliday junction recognition protein (HJURP), a centromere specific chaperone of CENP-A.^15^^,^^16^ The centromeric recruitment of Mis18 complex begins in late telophase and persists through early G1 which is critical for association of prenucleosomal HJURP/CENP-A/H4 complex to the centromeres.^17^ The Mis18 complex serves as a regulator for CENP-A/Cnp1 deposition in organisms containing regional centromeres.^14^^,^^16–21^

Cellular levels of Mis18 complex subunits are cell cycle regulated by β-transducin repeat containing protein (β-TrCP), a well-characterized substrate receptor of the E3 ligase SCF (Skp1, Cullin, F-box) complex.^22^ The SCF complex assembled with the F-box protein, β-TrCP (SCF^β-TrCP^) regulates the steady state levels of substrates through ubiquitination-mediated proteasomal degradation to maintain cellular homeostasis.^23–25^ A previous study has shown that HeLa cells depleted for β-TrCP showed reduced ubiquitination of Mis18β suggesting that β-TrCP regulates cellular levels of Mis18β.^22^ At the onset of mitosis, Mis18β along with Mis18α and Mis18BP1 forms a stable Mis18 complex, which recruits HJURP for CENP-A loading at centromeres.^17^^,^^22^

Given the essential role of CENP-A in chromosomal stability, in this study, we examined the cellular consequences of dysregulated Mis18β levels on chromosomal localization of CENP-A and chromosomal stability in a triple negative breast cancer cell line, MDA-MB-231. As reported previously for HeLa cells,^22^ our results showed that depletion of β-TrCP leads to defects in ubiquitination of Mis18β with increased steady state levels of Mis18β in MDA-MB-231 cells. Intriguingly, we also observed increased steady state levels of endogenous CENP-A in β-TrCP depleted cells. Mitotic chromosome spreads showed mislocalization of CENP-A to noncentromeric regions and CIN phenotypes such as increased incidence of micronuclei formation and chromosome segregation defects in β-TrCP depleted cells. Cells codepleted for β-TrCP and Mis18β showed reduced mislocalization of CENP-A. Consistent with these results, cells overexpressing Mis18β alone showed mislocalization of endogenous CENP-A and increased incidence of micronuclei. Our studies show that β-TrCP-mediated proteolysis of Mis18β prevents mislocalization of CENP-A and CIN.

## Results

### Defects in proteolysis of Mis18β in β-TrCP depleted cells

**Figure 1.**
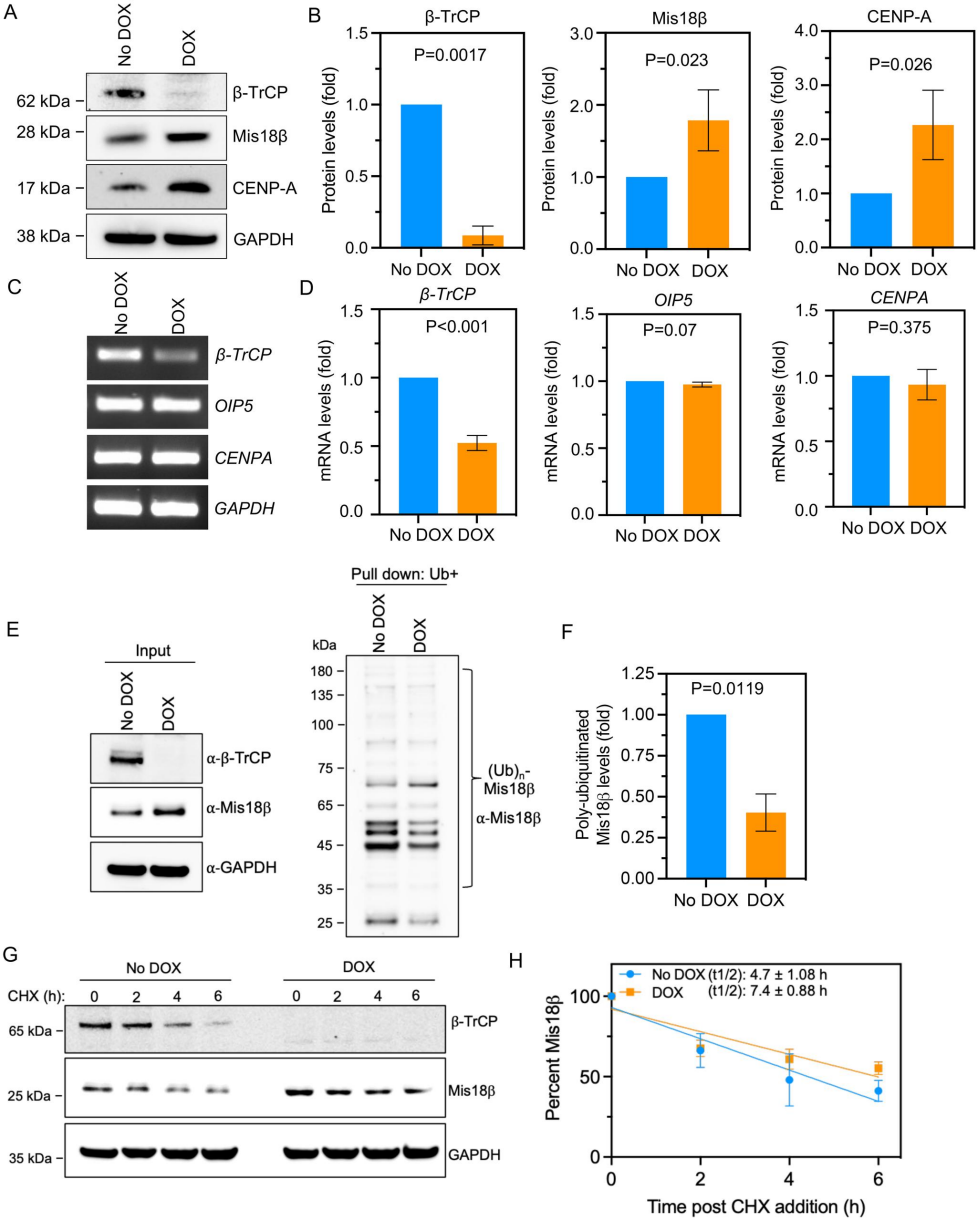
Protein levels but not the transcript levels of Mis18β are increased in β-TrCP depleted cells. (A and B) Western blots (A) and bar graphs (B) showing the protein levels of β-TrCP, Mis18β and CENP-A in MDA-MB-231^Δ β-TrCP^ cells untreated or treated with DOX for 48 h. The protein levels of Mis18β and CENP-A in B were calculated after normalization with GAPDH that was used as a loading control and expressed as fold increase relative to No DOX control. (C and D) Gel images from semi-quantitative RT-PCR (C) and bar charts from RT-qPCR (D) showing the mRNA levels of *β-TrCP*, *OIP5 (Mis18β)* and *CENPA* in MDA-MB-231^Δ β-TrCP^ cells untreated or treated with DOX for 48 h. The quantification in D was after the normalization with *GAPDH* that was used as an internal control and expressed as log2-fold difference. (E and F) Ubiquitin pulldown assay (E) showing the polyubiquitination of Mis18β in MDA-MB-231^Δ β-TrCP^ cells with or without DOX treatment and bar chart (F) depicting the polyubiquitinated Mis18β levels normalized against input Mis18β levels. (G and H) Protein stability assay using cycloheximide (CHX) depicting the stability of Mis18β in both DOX treated as well as untreated cells until 6 h and quantification plot (H) showing the percent Mis18β remaining in similar conditions. The half-life of Mis18β in both the conditions was calculated using GraphPad Prism. Error bars depict standard deviation (SD) from three biological repeats and the *P-*values were calculated using Student’s *t* test in B, D, F and H.

Centromeric localization of CENP-A is regulated by many factors and one of these is the Mis18 complex that comprises of Mis18α, Mis18β and Mis18BP1.^17^ β-TrCP, a substrate recognition subunit of an SCF ubiquitin ligase has been shown to regulate the proteolysis of Mis18β and this contributes to low levels of Mis18β in G1 and S phases in HeLa cells with ectopic expression of epitope tagged Mis18β.^22^ Given the role of Mis18β in loading CENP-A at centromeres,^17^ we investigated the consequences of deregulated Mis18β on chromosomal localization of endogenous CENP-A in β-TrCP depleted cells. We generated a triple negative breast cancer (TNBC) cell line, MDA-MB-231 that stably expresses doxycycline (DOX)-inducible shRNA against one of the two β-TrCP isoforms namely *β-TrCP1* referred to as MDA-MB-231^Δβ-TrCP^. Western blot analysis was used to examine the levels of β-TrCP, CENP-A and Mis18β, in MDA-MB-231^Δβ-TrCP^ cells treated with 1.0 µg/mL DOX for 48 h. Cells without DOX treatment were used as control. Western blot showed an efficient depletion of β-TrCP in MDA-MB-231^Δβ-TrCP^ cells treated with DOX (Figure 1A and B). As reported previously,^22^ we observed increased protein levels of Mis18β in DOX-treated MDA-MB-231^Δβ-TrCP^ cells (Figure 1A and B). Furthermore, a significant increase in protein levels of CENP-A was also observed in these cells (Figure 1A and B). To examine if higher protein levels of Mis18β and CENP-A were due to higher transcription, we used RT-PCR to quantify mRNA levels of *OIP5* (Mis18β) and *CENPA.* No significant difference in mRNA levels of *OIP5* or *CENPA* were observed in DOX-treated MDA-MB-231^Δβ-TrCP^ cells (Figure 1C and D). As expected, mRNA level of *β-TrCP* was significantly reduced in these cells. These results support a role for β-TrCP in regulating Mis18β at protein levels independent of an alteration at transcript levels. Next, we examined the ubiquitination status of Mis18β in cells with or without DOX treatment. A significant decrease (*P* = 0.0119) in polyubiquitination of Mis18β was evident in DOX treated condition compared to the no DOX control (Figure 1E and F). Consistent with the reduced ubiquitination of Mis18β, half-life of Mis18β was significantly increased (*P* = 0.027) from 4.7 ± 1.08 h to 7.4 ± 0.88 h in β-TrCP depleted cells as evident from the protein stability assay (Figure 1G and H). Based on these results, we conclude that defects in β-TrCP-mediated polyubiquitination of Mis18β contributes to increased steady state levels of Mis18β.

### β-TrCP depletion does not affect cell viability or cell cycle progression

The levels of Mis18β and CENP-A are cell cycle regulated and are increased in mitosis.^22^^,^^26^^,^^27^ Because we observed increased levels of Mis18β and CENP-A in β-TrCP depleted cells, we examined whether DOX-treatment for two days to deplete β-TrCP in MDA-MB-231^Δβ-TrCP^ cells affects proliferation and cell cycle progression. For cell proliferation assays, we counted total number of cells in control or β-TrCP-depleted MDA-MB-231^Δβ-TrCP^ cells. Our results showed that the total number of cells was comparable in control or β-TrCP depleted cells (Supplementary material, Figure S1A), indicating that proliferation of MDA-MB-231 cells is not affected upon β-TrCP depletion. For analysis of cell cycle progression, we examined nuclear morphology, performed flow cytometry, and assessed the levels of key cell cycle markers. In the first assay, we examined the nuclear morphology using DAPI staining to quantify cells in interphase, prometaphase, metaphase, anaphase, and cytokinesis. Our results showed no significant increase in the proportion of cells in a particular cell cycle stage upon β-TrCP depletion when compared to control cells (Supplementary material, Figure S1B). In the second assay, we used flow cytometry and observed that the cell cycle profiles are not altered in MDA-MB-231^Δβ-TrCP^ cells treated with DOX for two days, except for a mild increase (6%) in G1 population (Supplementary material, Figure S1C and D). In the third assay, Western blot analysis was used to examine the levels of cell cycle regulated proteins, cyclin E1 and phospho-histone H3S10 (pH3S10) which are generally higher in S and mitotic cell cycle stages, respectively. We observed that DOX-treated MDA-MB-231^Δβ-TrCP^ cells had reduced levels of β-TrCP confirming its efficient depletion and did not show significant changes in the levels of cyclin E1 or pH3S10 (Supplementary material, Figure S1E and F) showing that β-TrCP depletion does not arrest cells in a particular cell cycle stage. Taken together, our results showed that increased levels of Mis18β and CENP-A in β-TrCP depleted MDA-MB-231 cells are not due to defects in cell proliferation and cell cycle progression.

### Mislocalization of CENP-A to noncentromeric regions in β-TrCP depleted cells

**Figure 2.**
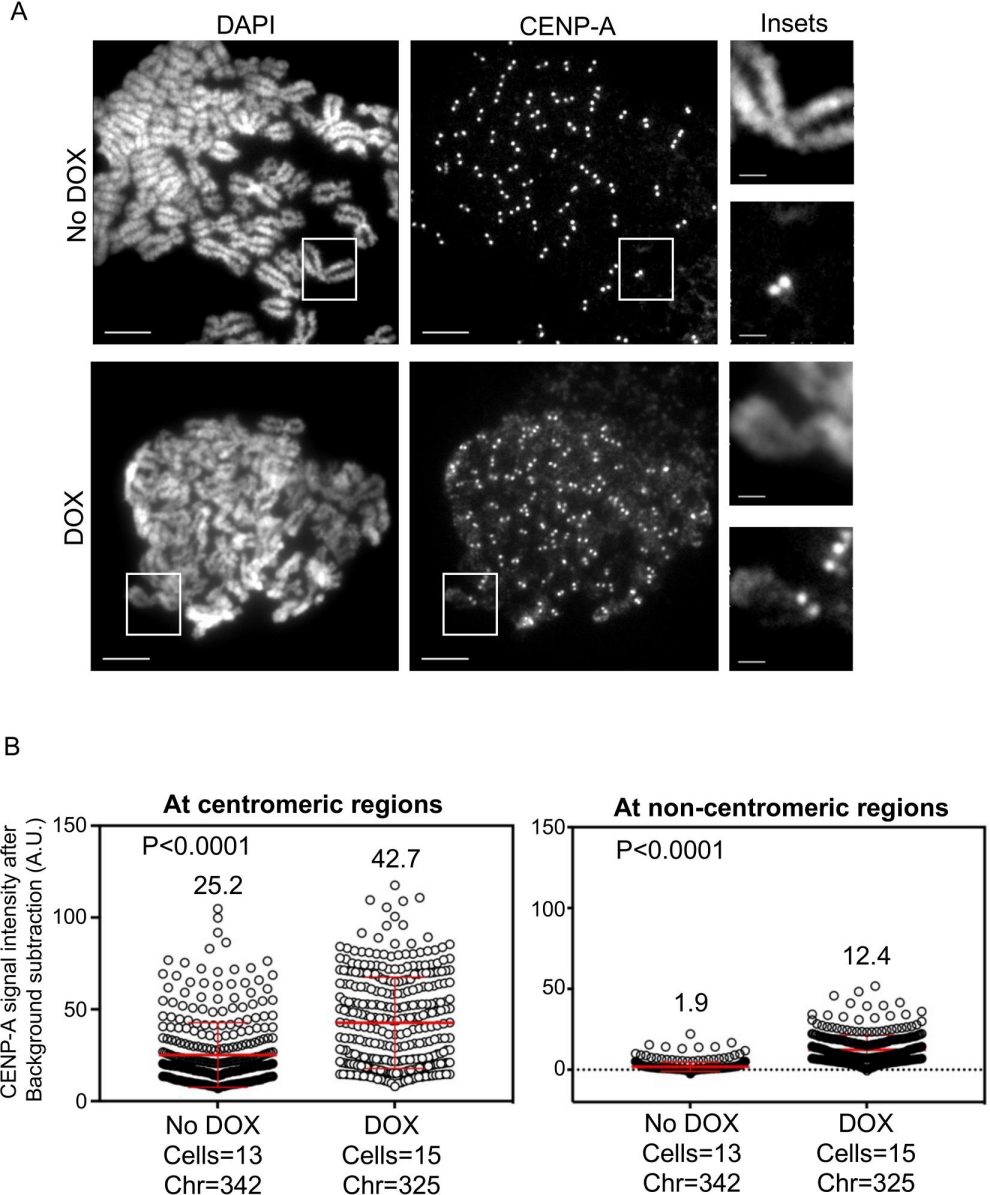
β-TrCP depletion contributes to CENP-A mislocalization in MDA-MB-231 cells. (A) Representative images of metaphase chromosome spreads prepared from MDA-MB-231^Δ β-TrCP^ cells untreated or treated with DOX for 48 h, showing the localization of endogenous CENP-A on mitotic chromosomes. Scale bars: 5 µm for main images, 2 µm for insets. (B) Quantification of CENP-A signal intensities (arbitrary units) at centromeric (left) and noncentromeric (right) regions in metaphase chromosome spreads of MDA-MB-231^Δ β-TrCP^ cells treated as in B. Each circle represents one spot quantified on chromosome. “Chr” represents number of chromosomes analyzed in the number of cells denoted. Error bars depict the SD across areas measured in the number of cells as indicated from three biological repeats and the *P-*values were calculated using Student’s *t* test.

Mis18α and Mis18β form a Mis18α/β heterodimer that promotes CENP-A deposition at centromeres through interaction between HJURP and Mis18β.^20^^,^^28^ Our results for increased levels of CENP-A in β-TrCP depleted cells prompted us to examine if this affects the chromosomal localization of endogenous CENP-A in these cells. The rationale for this is based on our studies describing a positive correlation between increased CENP-A levels and mislocalization of CENP-A to noncentromeric regions.^29–31^ Metaphase chromosomes spreads were prepared from control or DOX treated MDA-MB-231^Δβ-TrCP^ cells to examine the localization of endogenous CENP-A at centromeric and noncentromeric regions after immunostaining with anti-CENP-A antibody. The noncentromeric regions were defined as the regions on chromosome arms other than centromeres within the chromatids. We observed higher signal of CENP-A on chromosome arms in DOX treated cells compared to control cells (Figure 2A). Quantitative analysis of CENP-A signal intensities showed higher intensities of CENP-A at centromeric (1.7-fold) and noncentromeric regions (6.5-fold) in DOX treated MDA-MB-231^Δβ-TrCP^ cells when compared to control cells (Figure 2B). To confirm that the CENP-A mislocalization upon β-TrCP depletion is not specific to MDA-MB-231 cells only, we depleted β-TrCP using siRNAs in HEK293T cells (Supplementary material, Figure S2A) and used metaphase chromosome spread to examine CENP-A localization. Consistent with our results in MDA-MB-231 cells, CENP-A mislocalization to noncentromeric regions was increased (2.2-fold) in HEK293T cells upon β-TrCP depletion (Supplementary material, Figure S2B and C). Based on these results, we conclude that CENP-A is mislocalized to noncentromeric regions in β-TrCP depleted cells.

### β-TrCP depleted cells exhibit enhanced CIN phenotypes

**Figure 3.**
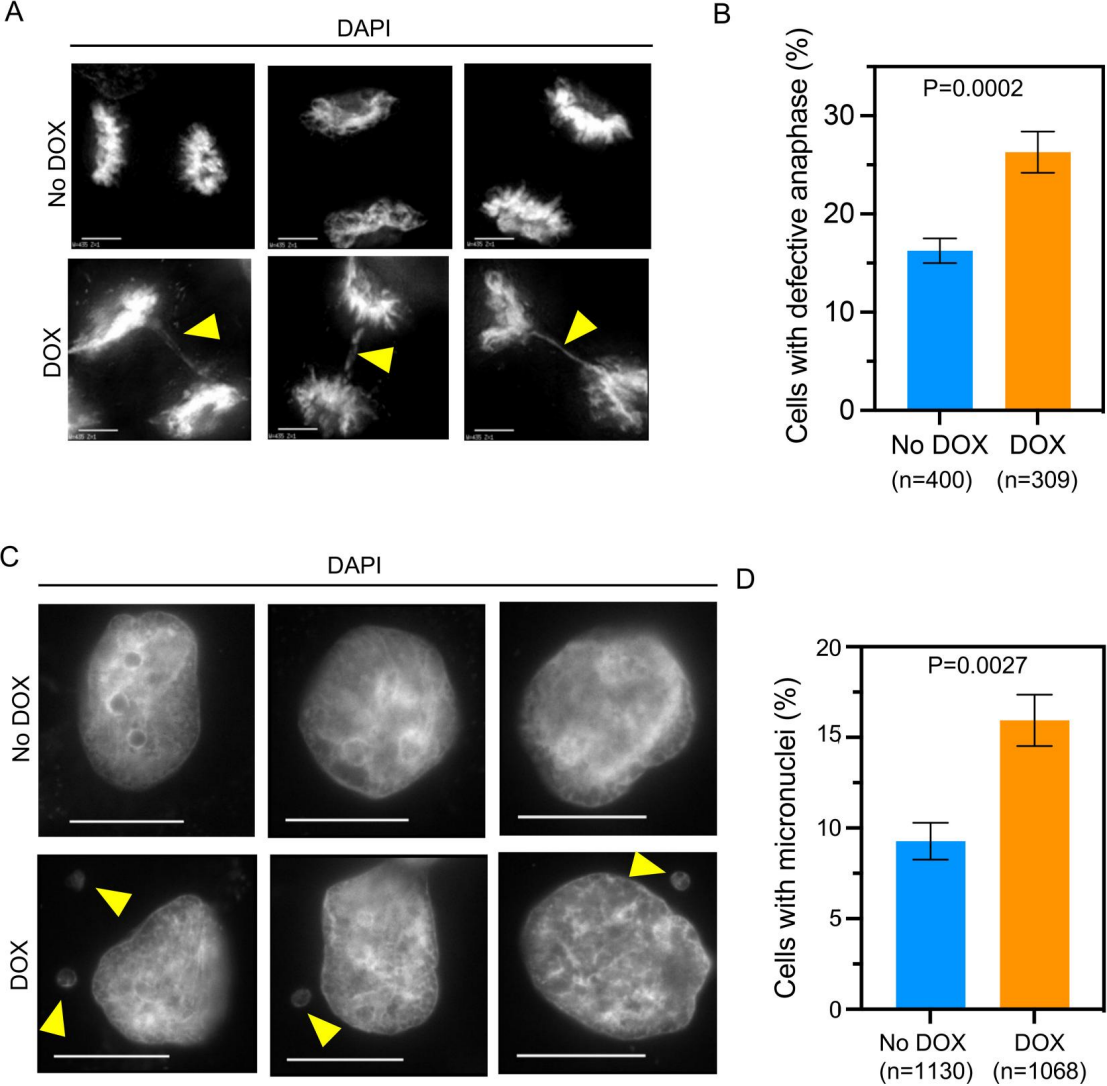
β-TrCP depletion contributes to chromosomal instability (CIN). (A) Representative DAPI stained images showing chromosome segregation status in MDA-MB-231^Δ β-TrCP^ cells untreated or treated with DOX for 48 h. Scale bar: 5 µm. The yellow arrowhead show lagging chromosomes and DNA bridges. (B) The bar graph depicting percent of cells with defective anaphase in MDA-MB-231^Δ β-TrCP^ cells treated as in A. (C) Representative DAPI stained images showing incidence of micronuclei in interphase as shown by yellow arrowheads in MDA-MB-231^Δ β-TrCP^ cells untreated or treated with DOX. Scale bar: 15 µm. (D) The bar graph depicting percent of cells with micronuclei in MDA-MB-231^Δβ-TrCP^ cells treated as in C. Error bars depict the SD across four and three biological repeats for graphs B and D, respectively. The *P-*values were calculated using Student’s *t* test in B and D.

Mislocalization of overexpressed CENP-A correlates with CIN phenotypes with increased errors in chromosome segregation and higher incidence of micronuclei in HeLa and DLD1 cells.^29–32^ We examined if the mislocalization of endogenous CENP-A in β-TrCP depleted MDA-MB-231 cells contributes to CIN phenotypes. Hence, control and DOX treated MDA-MB-231^Δβ-TrCP^ cells were stained with DAPI to examine defects in chromosome segregation (lagging chromosomes, uncongressed chromosomes and DNA bridges) and presence of micronuclei. We observed significantly increased chromosome segregation defects in DOX treated MDA-MB-231^Δβ-TrCP^ cells when compared to control cells (Figure 3A and B). Not surprisingly, control MDA-MB-231 cells also exhibited defective chromosome segregation due to its aneuploid characteristics (Figure 3B). Furthermore, significantly higher incidence of micronuclei was observed in DOX treated MDA-MB-231^Δβ-TrCP^ cells as compared to control cells (Figure 3C and D). Although chromosome segregation defects were observed even in control MDA-MB-231 cells, depletion of β-TrCP exacerbates the phenotypes in these cells indicating that β-TrCP depletion and CENP-A mislocalization contribute to increased CIN.

### Depletion of Mis18β suppresses mislocalization of CENP-A in β-TrCP depleted cells

**Figure 4.**
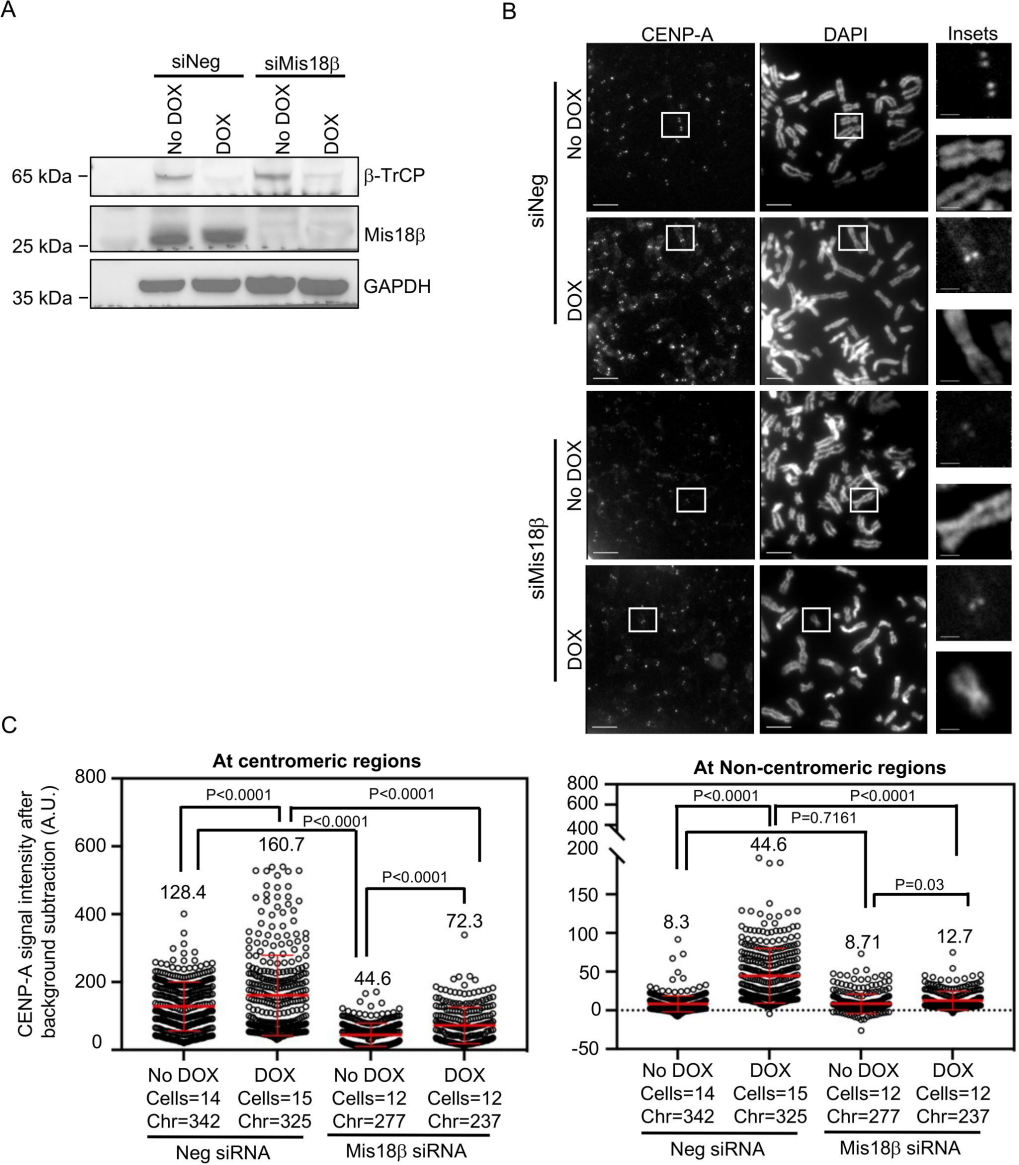
Co-depletion of β-TrCP and Mis18β prevents the mislocalization of CENP-A in MDA-MB-231^Δβ-TrCP^ cells. (A) Western blots showing the protein levels of β-TrCP and Mis18β in MDA-MB-231^Δβ-TrCP^ cells transfected with siRNAs as indicated for 72 h and untreated or treated with DOX for 48 h. GAPDH was used as a loading control. (B) Representative images of metaphase chromosome spreads prepared from MDA-MB-231^Δ β-TrCP^ cells transfected with siRNAs as indicated for 72 h and untreated or treated with DOX for 48 h showing the localization of endogenous CENP-A on mitotic chromosomes. Scale bar: 5 µm for main images and 2 µm for insets. (C) Quantification of CENP-A signal intensities (arbitrary units) at centromeric (left) and noncentromeric (right) regions in metaphase chromosome spreads of MDA-MB-231^Δ β-TrCP^ cells treated as in B. Each circle represents one spot quantified on chromosome. “Chr” represents number of chromosomes analyzed in the number of cells denoted. Error bars depict the SD across areas measured in the number of cells from three biological repeats and the *P*-values were determined using Student’s *t* test.

Increased levels of Mis18β and mislocalization of CENP-A upon β-TrCP depletion prompted us to examine whether elevated levels of Mis18β contribute to CENP-A mislocalization in β-TrCP depleted cells. To test this, we first transfected MDA-MB-231^Δβ-TrCP^ cells with siNeg or siMis18β for 72 h. Forty-eight hours prior to end of the experiment, DOX was added to the culture to deplete β-TrCP. Western blot analysis confirmed the depletion of β-TrCP in siNeg or siMis18β transfected cells treated with DOX (Figure 4A). Similarly, Mis18β was also efficiently depleted in MDA-MB-231^Δβ-TrCP^ cells transfected with siMis18β with or without DOX treatments (Figure 4A). Next, metaphase chromosome spreads were prepared from MDA-MB-231^Δβ-TrCP^ cells transfected with siNeg or siMis18β with or without DOX treatment. As expected, high intensities of CENP-A at noncentromeric regions were observed in siNeg transfected MDA-MB-231^Δβ-TrCP^ cells with DOX (Figure 4B and C). Quantitative analysis showed reduced CENP-A signal intensities at centromeric regions in MDA-MB-231^Δβ-TrCP^ cells transfected with siMis18β with or without DOX treatments (Figure 4C). This is consistent with a role for Mis18β in centromeric localization of CENP-A.^17^ The analysis further showed reduced CENP-A signal intensities at noncentromeric regions in DOX treated MDA-MB-231^Δβ-TrCP^ cells transfected with siMis18β , indicating that Mis18β depletion suppresses the mislocalization of endogenous CENP-A to noncentromeric regions in MDA-MB-231^Δβ-TrCP^ cells treated with DOX (Figure 4C, right panel). Based on these results, we conclude that Mis18β contributes to mislocalization of CENP-A in β-TrCP depleted cells.

### Overexpression of Mis18β alone contributes to CENP-A mislocalization and CIN phenotypes

**Figure 5.**
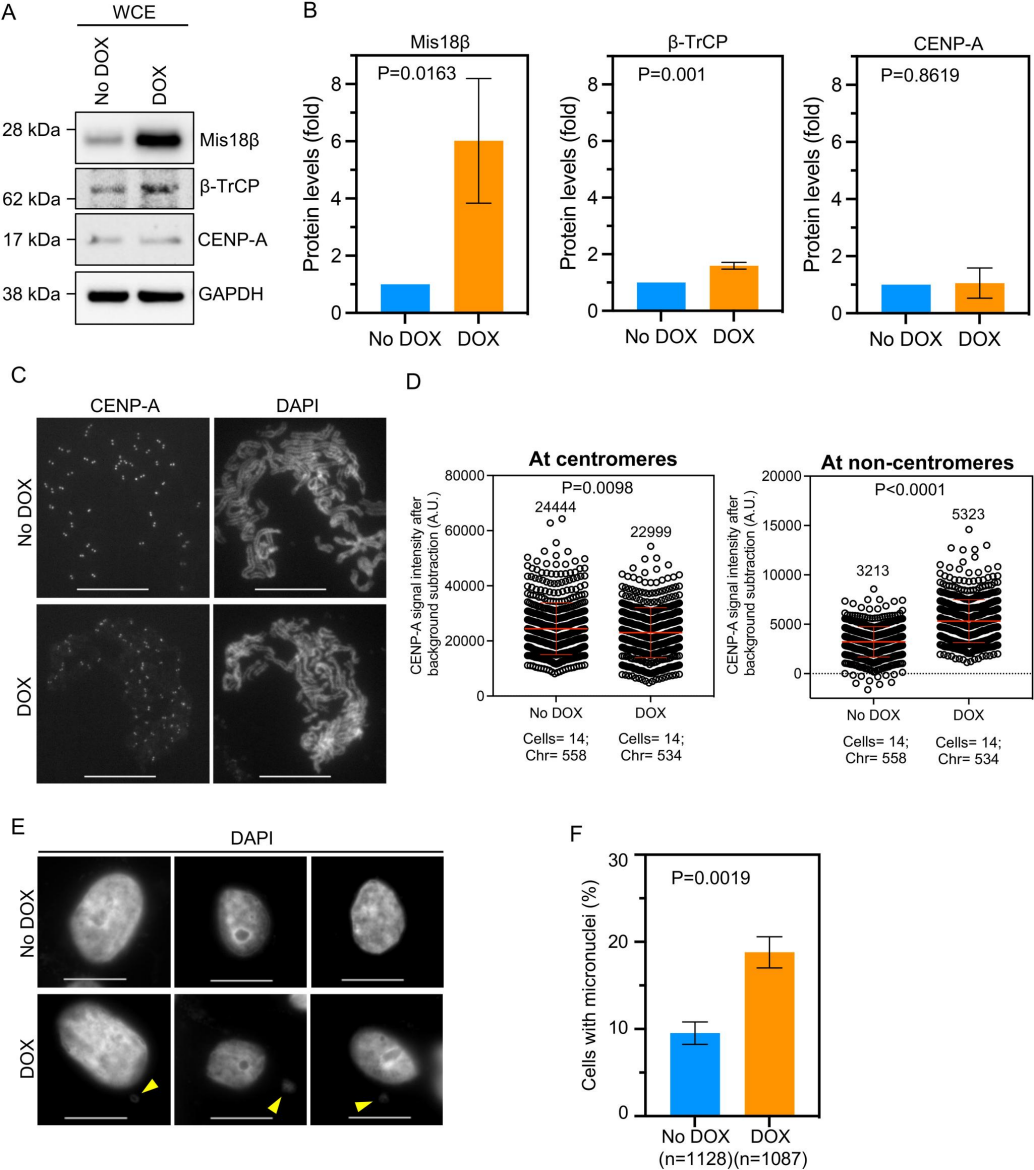
Overexpression of Mis18β alone contributes to CENP-A mislocalization in MDA-MB-231 cells. (A and B) Western blots (A) and bar graphs (B) showing the levels of proteins as indicated in MDA-MB-231^DOX-Mis18β^ cells treated or untreated with DOX for 48 h to overexpress Mis18β. The protein levels in B were calculated after normalization with GAPDH that was used as a loading control and expressed as fold increase relative to No DOX control. (C) Representative images of metaphase chromosome spreads prepared from MDA-MB-231^DOX-Mis18β^ cells untreated or treated with DOX for 48 h, showing the localization of endogenous CENP-A on mitotic chromosomes. Scale bar: 15 µm. (D) Quantification of CENP-A signal intensities (arbitrary units) at centromeric (left) and non-centromeric (right) regions in metaphase chromosome spreads of MDA-MB-231^DOX-Mis18β^ cells treated as in C. Each circle represents one spot quantified on chromosome. “Chr” represents number of chromosomes analyzed in the number of cells denoted. Error bars depict the SD across areas measured in the number of cells from three biological repeats. (E) Representative DAPI stained images showing incidence of micronuclei in interphase as shown by yellow arrow in MDA-MB-231^DOX-Mis18β^ cells untreated or treated with DOX. Scale bar: 15 µm. (F) The bar graph depicting percent of cells with micronuclei in MDA-MB-231^DOX-Mis18β^ cells treated as in E. Error bars depict the SD across three biological repeats and the *P-*values were calculated using Student’s *t* test in B, D and F.

Since our results suggested that elevated levels of Mis18β contributed to CENPA mislocalization in β-TrCP depleted cells, we proposed that overexpression of Mis18β alone might drive the mislocalization of CENP-A. To test this, we generated MDA-MB-231 cells stably overexpressing Mis18β in a DOX-inducible system (MDA-MB-231^DOX-Mis18β^). Western blots confirmed DOX-induced overexpression of Mis18β in MDA-MB-231^DOX-Mis18β^ cells treated with 1.0 µg/mL of DOX for 48 h (Figure 5A and B). While steady state levels of CENP-A were not significantly altered, a minor but significant increase in β-TrCP levels was observed in Mis18β overexpressed MDA-MB-231^DOX-Mis18β^ cells (Figure 5A and B), possibly as a feedback loop mechanism to regulate the levels of overexpressed Mis18β in these cells. To examine the localization of endogenous CENP-A, metaphase chromosomes spreads were prepared from MDA-MB-231^DOX-Mis18β^ cells with or without DOX treatment. Our results showed enhanced mislocalization of CENP-A on chromosome arms in DOX treated MDA-MB-231^DOX-Mis18β^ cells compared to control cells (Figure 5C). Quantitative analysis showed that the CENP-A signal intensities at noncentromeric regions were 1.7-fold higher in cells with Mis18β overexpression compared to control cells (Figure 5D). However, CENP-A signal intensities at the centromeric regions were modestly reduced by 1.06-fold in DOX-treated MDA-MB-231^DOX-Mis18β^ cells compared to the no DOX control (Figure 5D). One plausible explanation for the latter result is the titration of endogenous CENP-A to noncentromeric regions. Next, we tested whether Mis18β overexpression contributes to CIN phenotypes in MDA-MB-231 cells by staining control and DOX treated MDA-MB-231^DOX-Mis18β^ cells with DAPI and analyzed for incidence of micronuclei, one of the CIN phenotypes. A significantly higher incidence of micronuclei was observed in DOX treated MDA-MB-231^DOX-Mis18β^ cells compared to control cells (Figure 5E and F). Taken together, we conclude that Mis18β overexpression contributes to the mislocalization of endogenous CENP-A and increased incidence of micronuclei.

## Discussion

**Figure 6.**
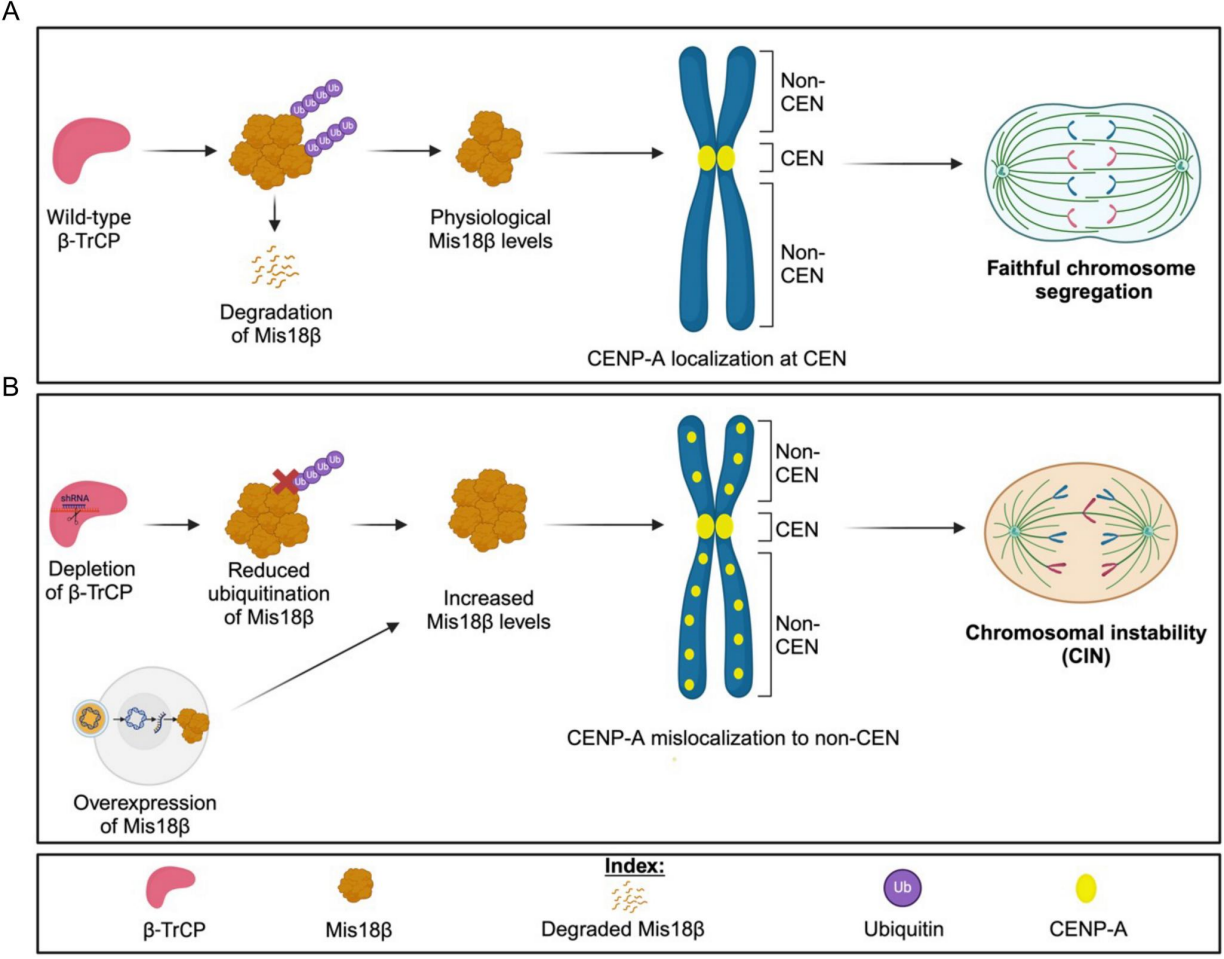
Proposed model depicting how β-TrCP depletion contributes to CIN. (A) β-TrCP being a substrate specifying factor for SCF E3 ubiquitin ligase recognizes Mis18β for ubiquitination and targets it for proteasomal degradation. The remaining physiological levels of Mis18β along with the other subunits of Mis18 complex helps in centromeric localization of CENP-A which ensures accurate chromosome segregation. (B) The steady state levels of Mis18β is increased in two ways, first with β-TrCP depletion and second with Mis18β overexpression. Such increased steady state levels of Mis18β contributes to noncentromeric localization of CENP-A and hence causes chromosomal instability.

The localization of CENP-A at the centromeres is essential for proper kinetochore assembly which facilitates stable kinetochore-microtubule attachments for faithful chromosome segregation. Overexpressed CENP-A mislocalizes to noncentromeric regions and this contributes to CIN phenotypes in yeasts, flies, human cells and xenograft mouse model.^29^^,^^31^^,^^33–36^ Investigation of mechanisms that prevent CENP-A mislocalization is an area of active study. Here, we defined a novel role for β-TrCP in preventing mislocalization of endogenous CENP-A to noncentromeric regions by regulating the levels of Mis18β, a licensing factor for centromeric CENP-A deposition.^20^ Cells depleted for β-TrCP show reduced ubiquitination, increased stability of Mis18β, mislocalization of CENP-A and CIN phenotypes. Furthermore, we show that Mis18β depletion suppresses CENP-A mislocalization observed upon β-TrCP knock-down, and overexpression of Mis18β alone enhances mislocalization of CENP-A to noncentromeric regions and CIN. We propose a model in which β-TrCP-mediated proteolysis of Mis18β prevents mislocalization of CENP-A for chromosomal stability. Deregulated Mis18β levels mediated by β-TrCP depletion or Mis18β overexpression promotes mislocalization of CENP-A and CIN (Figure 6).

We have defined an important regulatory role for posttranslational modification (PTM) of Mis18β through β−TrCP in the chromosomal localization of CENP-A. Overexpression of Mis18α/β was previously shown to increase centromeric CENP-A levels,^20^ however, the effects on CENP-A loading at noncentromeric regions had not been examined in these cells. Our results showed CIN phenotypes such as chromosome missegregation and higher incidence of micronuclei in β-TrCP depleted cells. β-TrCP is known to regulate the levels of spindle assembly checkpoint protein MAD2,^37^ and proteins related to centriole homeostasis,^38^ therefore, the CIN phenotypes observed upon β-TrCP depletion might not be exclusively due to CENP-A mislocalization. As we observed defects in ubiquitination of Mis18β and higher levels of endogenous CENP-A without affecting CENP-A transcription in β-TrCP depleted cells, we hypothesized that increased Mis18β levels contribute to the mislocalization of endogenous CENP-A. This is supported by increased noncentromeric CENP-A in β-TrCP-depleted cells, which is suppressed by Mis18β depletion, as well as by CENP-A mislocalization in cells overexpressing Mis18β. We propose that increased levels of endogenous CENP-A are due to mislocalization of CENP-A in β-TrCP-depleted cells. This is consistent with our observation for higher levels of endogenous CENP-A due to mislocalization of CENP-A in CHAF1B depleted cells.^30^ Previous studies have demonstrated roles for the histone chaperones such as HIRA, CHAF1B and the molecular co-chaperone DNAJC9 in preventing CENP-A mislocalization in human cancer cells lines.^30^^,^^32^ Taken together, these results show that deregulated expression of centromeric CENP-A loading factor such as Mis18β can promote mislocalization of CENP-A.

Our results underscore a noncanonical role for Mis18β in facilitating localization of CENP-A to noncentromeric regions by virtue of its deregulation. Other studies have shown that CENP-A deposition at centromeres is regulated by PTMs of CENP-A and its deposition factors. For example, phosphoregulation of HJURP and components of the Mis18 complex by the kinases PLK1 and CDKs are critical for the timely loading of centromeric CENP-A.^39^^,^^40^ This is particularly important as CENP-A expression, which occurs during G2/M, is uncoupled from its deposition at G1 in human cells.^13^^,^^27^ CDK1-mediated phosphorylation of HJURP has been shown to prevent its precocious targeting to the centromere.^41^ In *Xenopus* cells, CENP-A deposition is restricted during metaphase by phosphorylated HJURP along with the binding of Mis18BP1 to CENP-C.^42^ Phosphorylation of CENP-A at Serine 68 has been implied in preventing its interaction with HJURP and ubiquitin-linked degradation of CENP-A during mitosis.^43^^,^^44^ It has been proposed that impaired phosphoregulation of CENP-A promotes its mislocalization.^43^^,^^44^ We propose that increased Mis18β levels might be one of the pathways contributing to CENP-A mislocalization in β-TrCP depleted cells.

Our findings are clinically relevant as deregulated expression of all three proteins in this study, β-TrCP, Mis18β and CENP-A are implicated in cancers. For example, overexpression of Mis18β has been frequently observed in several cancer types including breast cancer, clear cell renal cell carcinoma, hepatic, gastric and colorectal cancers and this correlates with disease progression, poor prognosis, and low overall survival rate.^45–50^ A recent pan-cancer study showed that overexpressed *OIP5* (Mis18β) may contribute to tumor progression by increasing genome instability and affecting the tumor microenvironment.^51^ CENP-A overexpression has also been reported in many cancers including TNBC and is associated with poor clinical outcome for patients.^52–56^ Results from our in vitro studies using a TNBC cell line MDA-MB-231 suggests that elevated levels of Mis18β may serve as one of the key mechanisms that contributes to mislocalization of CENP-A and CIN. These studies provide important mechanistic insights into how increased expression of Mis18β (*OIP5*) and CENP-A may contribute to tumor progression and aneuploidy in human cancers.

## Materials and Methods

### Cell culture

All cell lines were cultured at 37 °C with 5% CO_2_ supply in Dulbecco’s modified Eagle’s Medium (DMEM) (12491023, Thermo Fisher Scientific) supplemented with 10% fetal calf serum (FCS) (Gibco-A3160501-50 mL), penicillin/streptomycin (15140122, Thermo Fisher Scientific), fungizone (15290018, Thermo Fisher Scientific), and L-glutamine (A2916801, Thermo Fisher Scientific). For frozen stocks, cells were mixed in freezing media (DMEM with 50% FCS and 5% DMSO) and stored at –80 °C. All cell lines were tested for mycoplasma-free status using the Universal mycoplasma detection kit (30-101K, ATCC) according to the manufacturer’s instructions.

### Plasmid construction and cell line generation

The pSLIK lentiviral vectors for depletion of β-TrCP were constructed as described previously.^57^ Briefly, oligonucleotides designed with *Bfu*AI compatible protrusion and containing the miR-shRNA sequence (5′- AGCGGCGTTGTATTCGATTTGATAATAGTGAAGCCACAGATGTATTATCAAATCGAATACAACGC-3′) were annealed and cloned into the *Bfu*AI site in pEN_TmiRc3 (Addgene plasmid #25748). The miR-shRNA sequence was designed by the RNAi codex algorithm (http://cancan.cshl.edu/RNAi_central/RNAi.cgi?type = shRNA). Lentiviruses were generated following recombination of pEN_TmiRc3 and pSLIK-PURO by transfecting HEK293T cells with the resulting recombination product together with packaging vectors pMDG, pCMVdeltaR8.91. pSLIK-PURO was generated from pSLIK-HYGRO (Addgene plasmid # 25737) by replacing the HYGRO cassette with the IRES-PURO cassette from pQXIP. Lentiviruses were collected 24- and 48-h posttransfection for target cell infection. The MDA-MB-231 cells were transfected with lentivirus and cells were selected with 1 µg/mL puromycin 48 h post viral infection to generate a stable pool of cells (MDA-MB-231^Δβ-TrCP^). For inducible Mis18β overexpression, Mis18β cDNA with sv40 polyadenylation signal sequence were PCR amplified from Mis18β expression vector (EX-T4051-M02, GeneCopoeia Inc.) and cloned into Gateway entry vector, pEN_TGmiRc3 cut with SacII and XbaI. The resulting Mis18β in the entry clone was then inserted under the DOX inducible promoter in lentiviral vector, pSLIK-HYGRO (Addgene) via Gateway cloning RL reaction. The Mis18β overexpression cell line (MDA-MB-231^DOX-Mis18β^) was generated by transducing the cells with the lentiviruses and the transduced cells post 48 h of viral infection were selected with 200 µg/mL hygromycin.

### siRNA transfections

The gene depletion experiments were carried out using individual siRNAs. The *Mis18β* (*OIP5*) siRNA was purchased from Sigma Aldrich (SASI_Hs01_00199026) whereas the custom *β-TrCP1* (AAGUGGAAUUUGUGGAACAUCUU) and nontargeting control pool (D-001810-10-20) siRNAs were ordered from Horizon Discovery. Following 48 h of seeding, the cells were forward transfected with 15 nM final concentration of siRNAs using RNAiMAX (13778150, Invitrogen) and Opti-MEM Reduced Serum Medium (31985070, Gibco) for 48 h. The cells were then used for various downstream assays.

### RT-PCR for transcript level analysis

The total RNA was extracted from cell pellets using the TRIzol reagent followed by purification with RNeasy Mini kit (Qiagen). The concentration of RNA was measured using NanoDrop spectrophotometer (Thermo Fisher) and 1 µg total RNA per sample was used for cDNA synthesis (Superscript IV Reverse transcriptase, Invitrogen). A concentration of 100 ng cDNA was used for each reaction. Following gene-specific primers were used for PCR: *β-TrCP* (FP:5′ CTGCAAGAGAAGGCACTCAA3′; RP: 5′GCCAGTCCCTGGGTTATACA3 ′), *OIP5* (FP:5′ TGGCATTGAAGGTTCACTCA3′; RP: 5′TTGTCACTGGAAAGGCAGAAG3′), *CENPA* (FP: 5′CGGAGACAAGGTTGGCTAAA3′; RP: 5′AGGCGTCCTCAAAGAGATGA3′), and *GAPDH* (FP: 5′ CTCTGCTCCTCCTGTTCGAC3′; RP: 5′TTAAAAGCAGCCCTGGTGAC3′). *GAPDH* expression was used as an internal control for this analysis. The PCR products were run on 2% agarose gels at 80 V for 60 min. The images were acquired using a gel documentation system (Bio-Rad) and the band intensity was quantified using the ImageJ software. The mRNA level expression of each gene in DOX-treated condition was calculated relative to the no DOX control.

### Propidium iodide (PI) staining for cell cycle analysis

Cells grown in the presence and absence of DOX were harvested following trypsinization and centrifuged at 500 *g* for 5 min. After washing the cell pellets twice with 1× PBS, the cells were then fixed with 70% ice-cold ethanol for 30 min at 4 °C. At the end of the fixation time point, cells were washed twice with 1× PBS followed by incubation with 20 µg/mL of RNaseA for 30 min at room temperature. A final concentration of 5 µg/mL PI was added to the cells. Flow cytometry was performed on a BD FACSymphony A5 instrument and data were analyzed using FlowJo (version 10.8.1).

### DAPI staining of cells for nuclear morphology and CIN phenotypes scoring

Cells were grown with and without DOX on glass coverslips in a 12-well cell culture dish. After 48 h of DOX induction, cells were fixed with pre-chilled methanol for 1 min followed by washing three times with 1× PBS, 5 min each at room temperature. Fixed cells were then stained with DAPI for 30 min at room temperature to visualize the nuclei, followed by washing three times with 1× PBS-T, 5 min each. The coverslips were mounted on microscopic slides using a mounting medium. Cells were then imaged under the Delta vision microscope on the DAPI channel (Ex: 360/40 nm; Em: 457/50 nm). Anaphase cells with normal or defective chromosome segregation, and interphase cells with or without micronuclei were quantified and recorded manually.

### Ubiquitin pull-down assay

The levels of polyubiquitinated endogenous Mis18β were examined using the ubiquitin pull-down assay as previously reported with minor modifications.^58^ The MDA-MB-231^Δβ-TrCP^ cells were grown in a 15 cm cell culture dish with and without DOX treatment for 48 h. Both no DOX and DOX treated cells were also incubated with the proteasome inhibitor MG132 (10 µM) for 4 h prior to harvesting. At the end of the timepoint, the cells were harvested using a scraper and were added with 0.5 mL of ubiquitin lysis buffer (20 mM Na_2_HPO_4_, 20 mM NaH_2_PO_4_, 5 mM tetra-sodium pyrophosphate, 50 mM sodium fluoride, 10 mM β-glycerophosphate, 2 mM EDTA, 5 mM *N*-ethylmaleimide, 1 mM DTT, 1 mM PMSF, 1% NP-40, and protease inhibitor cocktail; Sigma Aldrich, P8215). By using a bead beater (MP Biomedicals, FastPrep-24 5 G), the cells were lysed with Matrix C (MP Biomedicals) for 40 s. The cell lysates were collected in 1.5 mL microcentrifuge tubes and centrifuged at 6000 rpm for 5 min. The supernatant fractions were collected in fresh 1.5 mL MCTs, and the protein concentration of these fractions was measured using the DC protein estimation assay kit (Bio-Rad). Equal amount of proteins were taken for each condition and a total volume of 800 µL was made using the lysis buffer. From this, 40 µL of aliquot was taken out and considered as input. The remaining lysate was added to tubes containing the pre-equilibrated tandem ubiquitin-binding beads (Agarose-TUBE1, LifeSensors, UM401) and incubated overnight at 4 °C on a rotator. The beads were then washed three times with TBS-T at room temperature and the beads bound to the protein of interest were boiled with 60 µL of 2× Laemmli buffer at 95 °C for 10 min. Western blotting was done to detect the polyubiquitinated Mis18β by probing the nitrocellulose membranes with anti-OIP5 antibody (12142-1-AP, Proteintech). Similarly, the input samples were also probed for β-TrCP, Mis18β and GAPDH.

### Protein stability assay

The MDA-MB-231^Δβ-TrCP^ cells cultured in the presence or absence of DOX for 48 h were treated with cycloheximide (100 µg/mL) for 0, 2, 4, and 6 h. Whole cell lysates were prepared using Laemmli buffer at the end of each time point and the desired proteins were probed by immunoblotting.

### Immunoblotting

At the end of the desired time point, the cells were lysed with 2× Laemmli buffer followed by boiling at 95 °C for 10 min to prepare the samples for loading on SDS-PAGE gels. The primary antibodies as mentioned in the figures l were used at the indicated dilutions as follows: rabbit anti-β-TrCP (4394S, Cell Signaling Technology) at 1:500, rabbit anti-OIP5 at 1:500, mouse anti-CENP-A (ADI-KAM-CC006-E, ENZO) at 1:500, rabbit anti-cyclin E1 (mAb 20808, Cell Signaling Technology) at 1:1000 dilution, mouse anti-GAPDH (MA5-15738, Invitrogen) at 1:1000 and Rabbit anti-pH3S10 (06-570, Upstate) at 1:2000 dilution. HRP conjugated secondary antibodies against mouse (GENA931, Sigma) and rabbit (GENA934, Sigma) were used at 1:5000 dilution. Blots were incubated with SuperSignal West Pico PLUS chemiluminescent substrate (34578, ThermoFisher Scientific) prior to imaging using a Bio-Rad Imager. Image J was used to quantify signal intensities of bands from the blots.

### Mitotic chromosome spread preparation and immunofluorescence

Chromosome spreads were prepared for CENP-A localization studies as follows. At the 70% confluency, cells were treated with 200 ng/mL Colcemide (10295892001, Roche) for 5 h. The medium containing cells was collected first, and rest of the cells were trypsinized and centrifuged at 1200 rpm for 5 min at room temperature in 15 mL Falcon tubes. The cell pellets were resuspended with 1 mL of hypotonic solution (75 mM KCl) by adding in a dropwise manner, followed by mixing and incubating at 37 °C for 20 min. Cells were counted and resuspended to a concentration of 0.2 million cells/mL. A volume of 250 µL of cell suspension was cytospun at 900 rpm for 5 min. The cells were then hydrated with KCM buffer (10 mM Tris-HCl pH 8.0, 120 mM KCl, 20 mM NaCl, 0.5 mM EDTA, 0.1% Triton X-100), for 2 min, followed by permeabilization with KCM buffer with 0.5% Triton X-100 for 10 min. Cells were incubated with blocking solution (KCM + 1% BSA) for 30 min and incubated with mouse anti-CENP-A Ab (ADI-KAM-CC006-E, ENZO) as primary antibody for one hour at room temperature. After three washes for 5 min each with KCM buffer, the cells were incubated with goat anti-mouse DY 488 (35502, ThermoFisher Scientific) secondary antibodies (1:500 dilution), washed three times in KCM buffer. Cells were then fixed with 4% paraformaldehyde (28908, ThermoFisher Scientific) in KCM buffer for 15 min and washed with PBS, stained with DAPI for 10 min, washed twice with PBS + 0.1% Tween-20, followed by final wash with water and mounted on microscopic slides using the Prolong gold antifade mounting media containing DAPI (P36935, ThermoFisher Scientific).

### Microscopy and image acquisition

The images of immunostained cells were acquired using a Delta Vision Elite system (Applied Precision/Leica Microsystems, Inc., Deerfield IL) consisting of Olympus IX70 inverted microscope (Olympus America, Inc. Melville, NY) with 100X NA 1.4 oil immersion objective and a scientific CMOS camera controlled by *softWoRx* software. Filters used for imaging were FITC (Ex 475/28; Em 525/48), and DAPI (Ex 360/18; Em 435/48) with polychroic beam splitter (DAPI/FITC/RD/Cy5). Z-stacks of at least 10 focal planes were acquired with an exposure of 0.1 to 0.5 s, depending on the filter.

### Quantitative immunofluorescence analysis

To calculate the fluorescence intensities, boxes of 6 × 6 pixels were drawn on centromeric regions as defined by bright foci of CENP-A and on noncentromeric regions as defined by the signal outside the centromeric regions on a chromosome (chromosome spreads). Four boxes of 6 × 6 pixels were drawn at four random areas outside the chromosomes in the same cell for background correction. The data inspector tool in *softWoRx* was used to analyze the maximum intensity values from the regions of interest. Final fluorescence intensity for CENP-A was determined by subtracting the average background intensity. Intensity measurements were carried out for at least 10 centromeric and noncentromeric spots from each cell for an average of 10 cells from two to three independent experiments. Average values from more than 100 centromeric or noncentromeric spots were calculated and used for statistical analysis.

### Statistical analysis

The *P-*values were calculated using the unpaired *t* test (parametric) and two-way ANOVA using GraphPad Prism 10.

## Supplementary Material

Supplementary file_Sethi et al.docx

## Data Availability

The original data shown in the manuscript are available on Figshare (DOI: 10.6084/m9.figshare.25917475).
