# Supplementary material for: β-TrCP-Mediated Proteolysis of Mis18β Prevents Mislocalization of CENP-A and Chromosomal Instability
Source: Mol Cell Biol. 2024 Aug 13;44(10):429–42. doi: 10.1080/10985549.2024.2382445 (PMC11486186; doi:10.1080/10985549.2024.2382445)
Supplement: Supplementary file_Sethi et al.docx [file TMCB_A_2382445_SM2823.docx]

**Supporting Information**

**β-TrCP-Mediated Proteolysis of Mis18β** **Prevents Mislocalization of CENP-A and Chromosomal Instability**

Subhash Chandra Sethi^1,4^, Roshan Lal Shrestha^1,4^, Vinutha Balachandra^1^, Geetha Durairaj^2^, Wei-Chun Au^1^, Michael Nirula^1^, Tatiana S. Karpova^3^, Peter Kaiser^2^ and Munira A. Basrai^1*^

^1^Genetics Branch, National Cancer Institute, National Institutes of Health, Bethesda, MD 20892, USA.

^2^Department of Biological Chemistry, School of Medicine, University of California, Irvine, CA 92697, USA

^3^Laboratory of Receptor Biology and Gene Expression, National Cancer Institute, National Institutes of Health, Bethesda, MD 20892, USA.

^4^Equally contributing authors

*Address correspondence to: Munira A. Basrai ([basraim@nih.gov](mailto:basraim@nih.gov))

**Supplementary Figures:**

**Figure S1**

**Figure S2**


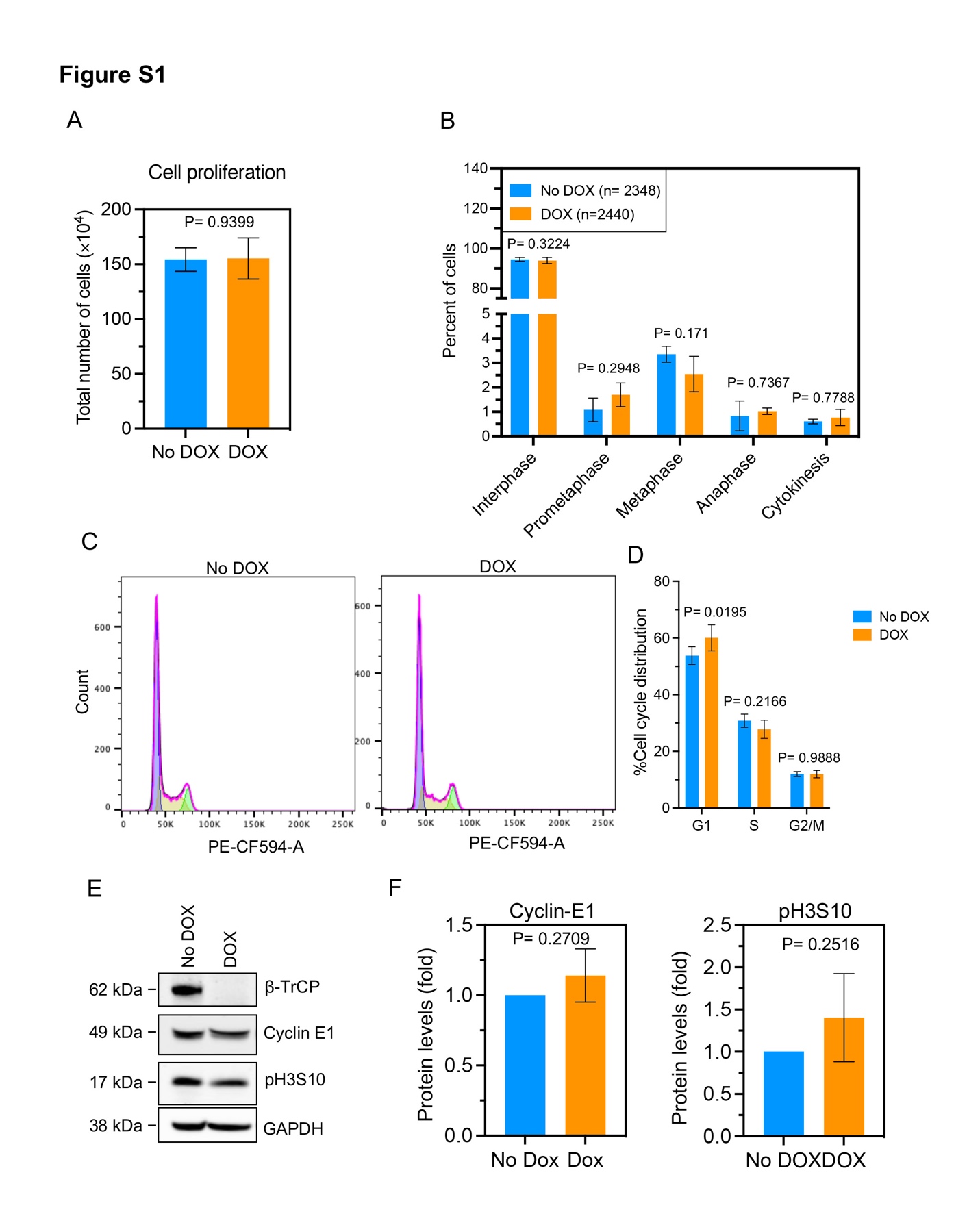


**Figure S1: β-TrCP depletion does not affect cell viability of cell cycle progression in MDA-MB-231 cells.** (A) Bar chart depicting the total number of cells in MDA-MB-231^Δ β-TrCP^ cells with and without DOX treatment after two days. (B) The proportion of MDA-MB-231^Δ β-TrCP^ cells in different cell cycle phases with and without DOX treatment are depicted as a bar chart. (C) Propidium Iodide (PI) staining followed by flow cytometry was done to analyze the cell cycle profiles in the cells depleted for β-TrCP. (D) The quantification of cells in different cell cycle stages is plotted from three independent experiments. (E) Western blots depicting the protein levels of β-TrCP, Cyclin E1 and pH3S10 in MDA-MB-231^Δ β-TrCP^ cells treated or untreated with DOX for 48 h. (F) The quantification plot of the indicated proteins is shown. For figures A and F, the error bars depict the SD across three biological repeats and the P-values were calculated using Student’s *t*-test. For figures B and D, error bars represent the SD across three biological repeats and the P-values were calculated using Two-way ANOVA with two-stage linear step-up procedure of Benjamini, Krieger and Yekutieli.


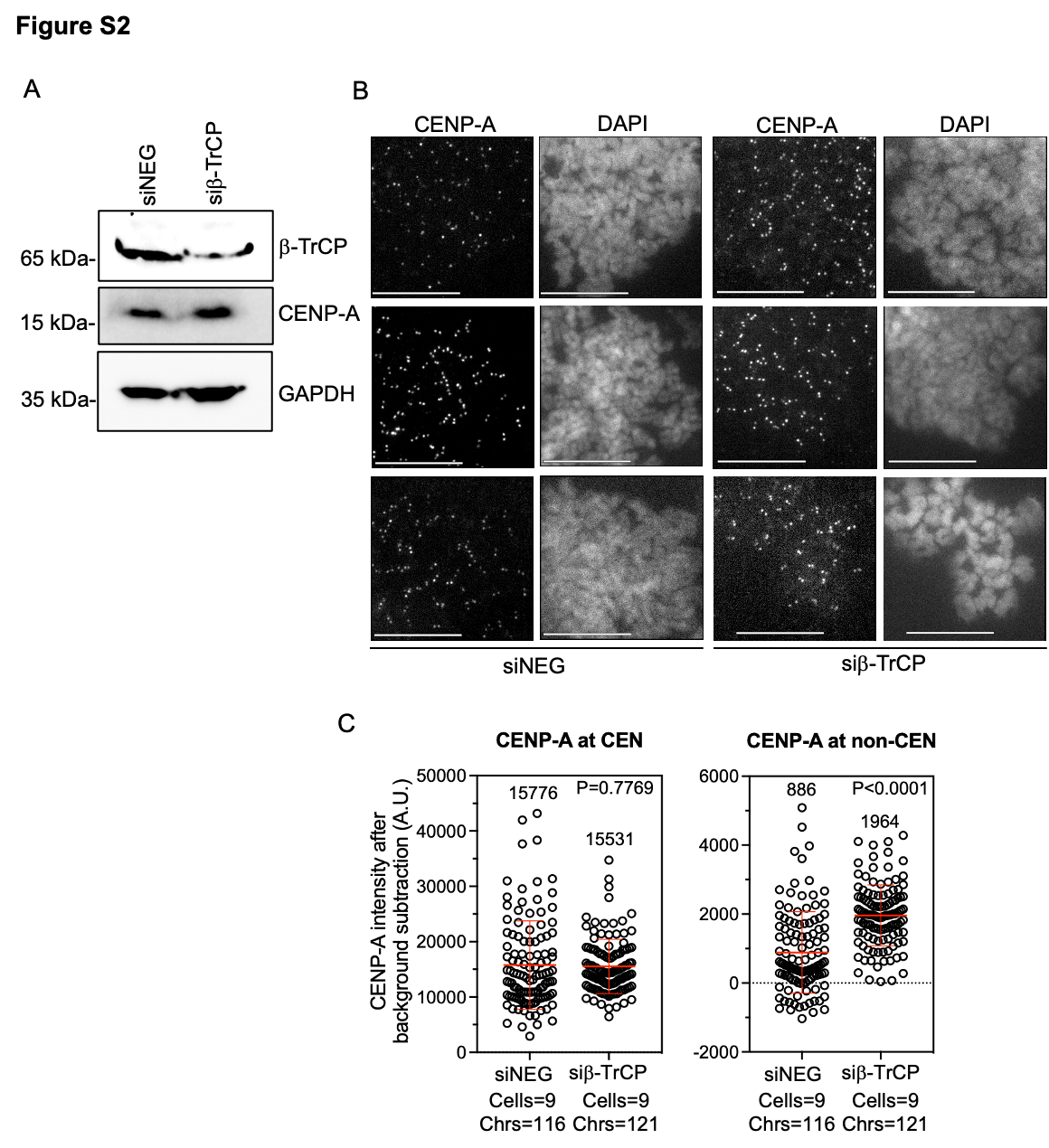


**Figure S2: Depletion of β-TrCP leads to mislocalization of CENP-A in HEK293T cells.** (A) Western blots depicting the protein levels of β-TrCP and CENP-A in HEK293T cells transfected with indicated siRNAs for 72 h. GAPDH was used for loading control. (B) Representative images of mitotic chromosome spreads showing the localization of endogenous CENP-A on mitotic chromosomes prepared from HEK293T cells following transfection with both siNEG and siβ-TrCP siRNAs. Scale bar: 15 μm. (C) CENP-A signal intensities (arbitrary units) at centromeric (left) and non-centromeric (right) regions in metaphase chromosome spreads of HEK293T cells were quantified and plotted as scatter plots. Each circle denotes one spot quantified on chromosome. “Chr” represents number of chromosomes analyzed in the number of cells mentioned. Error bars depict the SD across areas measured in the number of cells from three independent repeats. The statistical significance was calculated using Unpaired t-test.
